# Supplementary material for: Elution Kinetics from Antibiotic-Loaded Calcium Sulfate Beads, Antibiotic-Loaded Polymethacrylate Spacers, and a Powdered Antibiotic Bolus for Surgical Site Infections in a Novel In Vitro Draining Knee Model
Source: Antibiotics (Basel). 2021 Mar 8;10(3):270. doi: 10.3390/antibiotics10030270 (PMC8000420; doi:10.3390/antibiotics10030270)
Supplement: Supplementary file 1 [file antibiotics-10-00270-s001.zip › supplementary/antibiotics-1120192-supplementary-proof.docx]

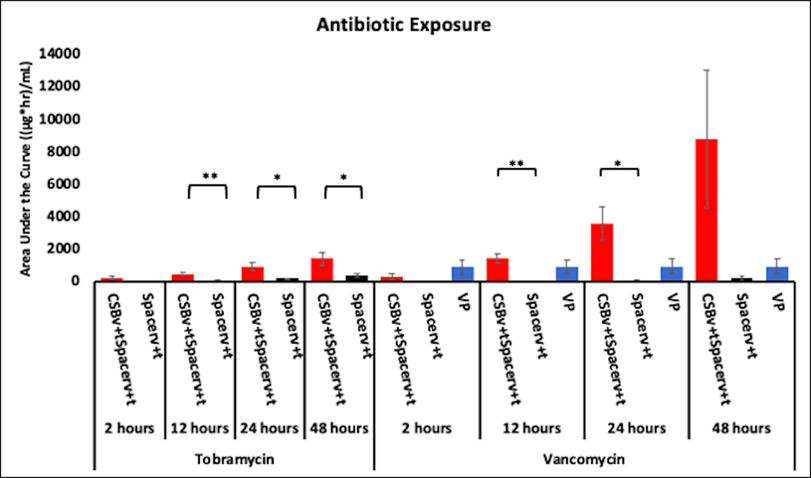


**Figure S1.** Tobramycin and vancomycin exposure via antibiotic administration methods to the system at 2, 12, 24 and 48 hrs. N= 4 replicates for Spacerv+t and CSBv+tplusSpacerv+t and 3 for VP; mean ± SE. (* P < 0.05, ** P < 0.01).
